# 

**Published:** 1884-03

**Authors:** 


### Article VIII.

### The Next Meeting of the British Medical Association.

The fifty-second annual meeting of the British Medical Association will be held on July 29, 30 and 31, 1884, at Belfast, under the presidency of James Cuming, M.A., M.D., F.K.Q.C.P.I., Professor of Medicine, Queen's College, Belfast.

The address in Medicine will be delivered by Sir Andrew Clark, Bart., M.D., F.R.C.P., Physician and Lecturer on Clinical Medicine, London Hospital.

The address in Obstetric Medicine will be delivered by Geo. H. Kidd, M.D., F.R.C.S.I., Master of the Coombe Lying-in Hospital, Dublin.

The address in Physiology will he delivered by Peter Redfern, M.D., F.R.C.S.E., Professor of Anatomy and Physiology, Queen's College, Belfast.

Visitors from America to attend this meeting can travel by any of the following routes:

1. A "Cunard" steamer will leave (*a*) New York on Wednesday, July 16, arriving in Queenstown about the following Thursday week, July 24. (*b*) Boston on Saturday, July 19, reaching Queenstown the following Monday week, July 28.

2. A "White Star" steamer will leave New York on Saturday, July 12, and on Saturday, July 19; due at Queenstown about July 20, and July 27.

3. An "Inman" steamer will leave New York on Tuesday, July 15; due at Queenstown about July 23.

4. An "Allan" steamer will leave Quebec on Saturday, July 19, arriving in Londonderry about the 26th or 28th of July.

5. An "Anchor" steamer will leave New York on Saturday, July 19; due at Londonderry on July 29.

Londonderry is 95 miles from Belfast, and trains run daily between the two places.

The route from Queenstown to Belfast is from Queenstown to Cork, Cork to Dublin, (165 miles by train), and Dublin to Belfast, (113 miles).

